# Hexamerization: explaining the original sin of IgG-mediated complement activation in acute lung injury

**DOI:** 10.1172/JCI181137

**Published:** 2024-06-03

**Authors:** Hrishikesh S. Kulkarni

**Affiliations:** Division of Pulmonary and Critical Care Medicine, John T. Milliken Department of Medicine, Washington University School of Medicine, St. Louis, Missouri, USA.

## Abstract

Although antibody-mediated lung damage is a major factor in transfusion-related acute lung injury (ALI), autoimmune lung disease (for example, coatomer subunit α [COPA] syndrome), and primary graft dysfunction following lung transplantation, the mechanism by which antigen-antibody complexes activate complement to induce lung damage remains unclear. In this issue of the *JCI*, Cleary and colleagues utilized several approaches to demonstrate that IgG forms hexamers with MHC class I alloantibodies. This hexamerization served as a key pathophysiological mechanism in alloimmune lung injury models and was mediated through the classical pathway of complement activation. Additionally, the authors provided avenues for exploring therapeutics for this currently hard-to-treat clinical entity that has several etiologies but a potentially focused mechanism.

## Antibody-mediated lung damage

**Figure 1:**
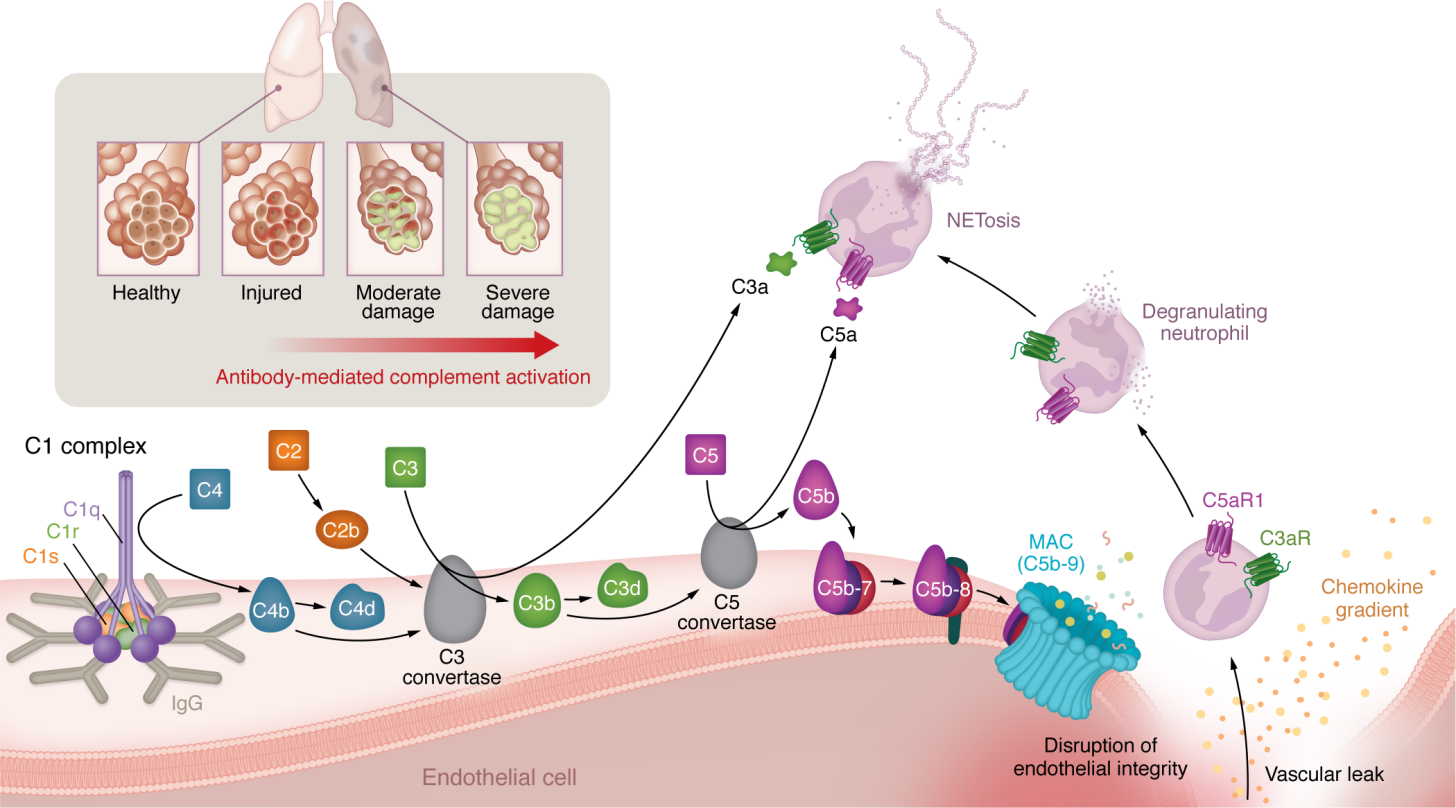
Alloantibodies induce complement-mediated lung injury. C1q binds to the Fc region of IgG hexamers on a pulmonary endothelial surface, resulting in the activation and assembly of two copies each of the serine proteases C1r and C1s, thus forming a C1 complex. The C1 complex cleaves C4 to C4b on a surface, which is processed to C4d and remains covalently bound. C4b, along with C2b, forms a C3 convertase. C3 convertase cleaves C3, releasing C3a into the fluid phase, while C3b and C3d remain bound to the cell surface, amplifying the cascade and forming the C5 convertase, which eventually facilitates the formation of the MAC (aka C5b-9). MAC disrupts endothelial integrity, resulting in vascular leak. At the same time, chemokines and anaphylatoxins, such as C3a and C5a, result in neutrophil activation. Subsequent neutrophil degranulation and the formation of NETs perpetuate injury. This ongoing injury results in moderate-to-severe lung damage, which, in its worst form, is characterized by histologic evidence of injury (such as septal thickening and hyaline membranes in humans), pulmonary inflammation, alveolar-capillary barrier disruption, and physiologic dysfunction, such as impaired gas exchange.

Surface-bound antigen-antibody complexes can initiate the proteolytic cascade of complement by binding to and activating the C1 complex, which is known as the classical pathway of complement activation (1). Antibody-dependent complement activation requires binding of one Fab arm to the target with the other remaining free, thereby allowing the formation of IgG hexameric Fc platforms (2). IgG subclass members IgG1 and IgG3 and IgM are known to activate complement via C1q binding to their Fc region (3). IgA lacks a site for C1q binding but is still able to activate the complement cascade via the mannose-binding lectin pathway (4). Binding of C1q to a surface results in the activation and assembly of two copies each of the serine proteases C1r and C1s (5), which can cleave C4 to C4b (and C4a), and C2 to C2b (and C2a), thus forming the C3 convertase (C4bC2b, formerly referred to as C4bC2a) (6). The C3 convertase cleaves C3, which generates C3a, an anaphylatoxin that is also a potent inflammatory mediator (7). Importantly, addition of a C3b to the C3 convertase forms the C5 convertase, leading to a membrane attack complex (MAC) that can perturb membranes and result in cell lysis (Figure 1) (8).

Although antibody-mediated lung damage is a major contributing factor in transfusion-related acute lung injury (TRALI) (9), autoimmune lung disease (for example, coatomer subunit α [COPA] syndrome) (10), as well as primary graft dysfunction (PGD) occurring after lung transplantation (11), the mechanism by which antigen-antibody complexes activate the complement system to induce lung damage remains unclear. A theory that has increasingly gained traction involves the formation of IgG hexamers from IgG1 and IgG3 antibodies on antigenic surfaces by Fc-domain interactions that result in complement activation and increased deposition on these target surfaces (2, 12). Whether this hexamer assembly explains alloantibody-mediated lung damage in vivo has been an unanswered question in the field.

## Antibody hexamerization in complement-mediated alloimmune acute lung injury

In this issue of the *JCI*, Cleary et al. utilized several approaches to demonstrate that IgG forms hexamers with MHC-I Class I alloantibodies, and this hexamerization is required for complement-mediated alloimmune acute lung injury (ALI) (13). They used multiple orthogonal approaches to block hexamerization, such as antibody carbamylation, the knockin mutation K439E in the Fc region, and a treatment with domain B from Staphylococcal protein A, all of which reduced alloantibody-mediated ALI. Conversely, mutations in the Fc region that promoted hexamerization worsened this phenotype. Utilizing C1q-deficient mice, the authors demonstrated a key role of the classical pathway of complement in the pathophysiology of their model. Additionally, they pinpointed the specific location of antibody-mediated complement deposition, specifically on the pulmonary endothelium. Finally, in an elegant lung injury model involving the administration of 34-1-2S, a monoclonal antibody that preferentially binds MHC class I antigens, the authors demonstrated a causal relationship between IgG hexamerization and lung damage. They administered 34-1-2S to transgenic mice expressing human FCGR2A, and then used a recombinant Fc hexamer “decoy” therapeutic. This study, therefore, convincingly demonstrates that IgG hexamerization is required for alloantibody-mediated ALI. Additionally, the study provides unique therapeutic avenues for an otherwise hard-to-treat critical illness, the current treatment of which is primarily supportive. Thus, the study by Cleary et al. (13) not only provides a unique opportunity to reflect on our current understanding of alloantibody-mediated lung injury, but also generates key questions as to how we may be able to design subsequent studies to translate therapeutics from preclinical models of complement-dependent alloimmune lung injury to patient care.

## The time course of complement activation in alloimmune ALI

Given that IgG hexamerization induces alloantibody-mediated lung damage via activation of the classical pathway of complement, understanding the time course for initiation of complement activation and injury is important in this disease. The pathophysiology of the ALI model used by Cleary et al. has two major components — an early component, occurring within minutes, related to intravascular platelet and neutrophil responses that can limit lung microvascular perfusion, and a following, later component involving inflammatory injury of the endothelium that increases lung vascular permeability (13). The authors have previously shown that complement activation, as demonstrated by C3 deposition, occurs within five minutes of the inciting event, with complement-dependent intravascular platelet and neutrophil responses observed within five to ten minutes of 34-1-2S injection (14). The work by Cleary et al. advances our understanding of the pathophysiology of alloantibody-mediated damage, in that pulmonary edema was detectable around 15 minutes after 34-1-2S injection, which follows the onset of intravascular platelet and neutrophil responses (13). Classical complement activation continued for several hours after the inciting event, and its footprint remained even after the vascular permeability had resolved (e.g., at 24 hours) (13), which is consistent with the hallmarks of a different disease, C4d-positive, antibody-mediated rejection (AMR) following lung transplantation (15). Yet, susceptible hosts (in this case, h*FCGR2A*-transgenic mice) became more hypoxemic than did their littermates in the setting of alloantibody-mediated damage and often died before pulmonary edema developed, suggesting that limiting microvascular lung perfusion is a key component of this model. Moreover, these susceptible hosts often had increased leukocyte recruitment into their lungs, and thus the survivors may have developed worse pulmonary edema (13).

Therefore, this model of alloimmune lung damage — occurring within minutes to hours of an 34-1-2S injection — creates an opportunity to investigate not only when complement activation occurs, but also when it terminates. The determining factors of its termination (for example, regulatory proteins), its interplay with neutrophil recruitment and alveolar-capillary barrier disruption, and how complement ties into the resolution of alloimmune lung injury are questions requiring further attention. Addressing these questions may help determine the optimal timing and target for therapies. For example, would it be better to target canonical proteins such as C1q or C5 in alloimmune lung injury, as has previously been done in AMR (16, 17)? And until when would this approach be effective, compared with using a strategy that interferes with antibody binding (for example, intravenous Ig [IVIg] therapy) in combination with plasmapheresis to clear out preexisting antibodies until antibody-depleting therapies kick in (18)? Alternatively, should we work toward upregulating or delivering complement regulatory proteins locally to accelerate the termination of complement activation (19, 20)? Finally, given that neutrophil recruitment was increased in this model (13), neutrophil degranulation and neutrophil extracellular trap–mediated (NET-mediated) damage is a feature of TRALI (21), and C5aR1 signaling via complement cascade activation drives lung injury through NETs (22), this study, along with others, creates a precedent to interrogate whether complement modulation would affect factors inciting alloimmune ALI.

## Interfering with alloimmune ALI

Another important contribution of Cleary et al. are the methods by which hexamerization were inhibited to abrogate complement-mediated alloimmune ALI. There are several caveats that need to be considered as such therapeutics emerge from preclinical models into the clinical arena. First, the authors appropriately used multiple doses of human IgG1-34-1-2S to develop a model of sublethal ALI in susceptible mice. Subsequently, they randomized mice to receive CSL777, SCIg, or vehicle controls prior to injection with 34-1-2S. CSL777 is an investigational recombinant Fc hexamer serving as a “decoy” treatment that inhibits classical complement activation. It was based on a structure, known as entry 7X13, in the experimental Protein Data Bank (23). SCIg is a human plasma–derived immunoglobulin product that is currently used to treat antibody-mediated diseases. This approach allowed the authors to test how pulmonary complement deposition, and thus activation, was affected in their model. CSL777 resulted in considerably lower pulmonary endothelial C4b/d deposition compared with SCIg treatment. Notably, the response to SClg treatment was similar to IVIg therapy, which at present is the most widely used approach to inhibit antigen-antibody complex formation.

However, an important aspect of any such study is to define what is truly ALI. Historically, defining whether ALI has occurred in a model system ideally requires fulfillment of at least three of four domains: histological evidence of injury, presence of lung inflammation, alveolar-capillary barrier disruption, and/or evidence of physiological dysfunction (24). More recently, the field has evolved to acknowledge that experimental ALI encompasses a continuum of models ranging from those focusing on gaining specific mechanistic insights to those primarily concerned with preclinical testing of therapeutics or interventions (25). Thus, although mechanistic studies may justifiably focus on a single domain of lung injury, in order to increase the chance of success of clinical translation, it would be helpful to investigate the effects of a therapeutic on multiple domains. In Cleary et al., a considerable number of readouts focused on lung vascular permeability and excess lung water, although evidence of lung inflammation (e.g., neutrophil staining) and physiological readouts of impaired gas exchange were demonstrated in certain scenarios (13). As the goal of experimental modeling advances from the study of basic mechanisms to preclinical drug testing in antibody-mediated alloimmune lung injury and the need to ensure translation to clinical studies increases, there may be merit in demonstrating alterations of all four domains using measures that are considered relevant and potentially implementable by investigators across the globe, potentially even in resource-limited settings.

## Conclusions

In summary, the work by Cleary and colleagues makes seminal contributions to the field by demonstrating that hexamerization of IgG is a key pathophysiological mechanism of alloimmune lung injury and is mediated through the classical pathway of complement activation. Additionally, it provides avenues for exploring therapeutics to target this currently hard-to-treat clinical entity that has several etiologies but a potentially focused mechanism. The field of lung injury eagerly awaits such personalized therapeutics to mitigate the short-term and long-term consequences of acute respiratory distress syndrome, as we begin to better understand many of its etiologies.

## Version 1 06/03/2024

Electronic publication

## References

[1] Sahu SK (2022). Emerging roles of the complement system in host-pathogen interactions. Trends Microbiol.

[2] Diebolder CA (2014). Complement is activated by IgG hexamers assembled at the cell surface. Science.

[3] Sharp TH (2019). Insights into IgM-mediated complement activation based on in situ structures of IgM-C1-C4b. Proc Natl Acad Sci U S A.

[4] Roos A (2001). Human IgA activates the complement system via the mannan-binding lectin pathway. J Immunol.

[5] Almitairi JOM (2018). Structure of the C1r-C1s interaction of the C1 complex of complement activation. Proc Natl Acad Sci U S A.

[6] Kemper C (2014). Complement nomenclature 2014. Mol Immunol.

[7] Kulkarni HS (2018). The complement system in the airway epithelium: An overlooked host defense mechanism and therapeutic target?. J Allergy Clin Immunol.

[8] Menny A (2018). CryoEM reveals how the complement membrane attack complex ruptures lipid bilayers. Nat Commun.

[9] Strait RT (2011). MHC class I-specific antibody binding to nonhematopoietic cells drives complement activation to induce transfusion-related acute lung injury in mice. J Exp Med.

[10] Watkin LB (2015). COPA mutations impair ER-Golgi transport and cause hereditary autoimmune-mediated lung disease and arthritis. Nat Genet.

[11] Yang W (2022). IL-1β-dependent extravasation of preexisting lung-restricted autoantibodies during lung transplantation activates complement and mediates primary graft dysfunction. J Clin Invest.

[12] Abendstein L (2023). Complement is activated by elevated IgG3 hexameric platforms and deposits C4b onto distinct antibody domains. Nat Commun.

[13] Cleary SJ (2024). IgG hexamers initiate complement-dependent acute lung injury. J Clin Invest.

[14] Cleary SJ (2020). Complement activation on endothelium initiates antibody-mediated acute lung injury. J Clin Invest.

[15] Kulkarni HS (2015). Antibody-mediated rejection in lung transplantation. Curr Transplant Rep.

[16] Parquin F (2020). C1-esterase inhibitor treatment for antibody-mediated rejection after lung transplantation: two case reports. Eur Respir J.

[18] Vacha M (2017). Antibody depletion strategy for the treatment of suspected antibody-mediated rejection in lung transplant recipients: Does it work?. Clin Transplant.

[19] Li C (2021). A novel injury site-natural antibody targeted complement inhibitor protects against lung transplant injury. Am J Transplant.

[20] Vittal R (2022). Overexpression of decay accelerating factor mitigates fibrotic responses to lung injury. Am J Respir Cell Mol Biol.

[21] van der Velden S (2024). Complement activation drives antibody-mediated transfusion-related acute lung injury via macrophage trafficking and formation of NETs. Blood.

[22] Silva BM (2023). C5aR1 signaling triggers lung immunopathology in COVID-19 through neutrophil extracellular traps. J Clin Invest.

[23] Zuercher AW (2019). Next-generation Fc receptor-targeting biologics for autoimmune diseases. Autoimmun Rev.

[24] Matute-Bello G (2011). An official American Thoracic Society workshop report: features and measurements of experimental acute lung injury in animals. Am J Respir Cell Mol Biol.

[25] Kulkarni HS (2022). Update on the features and measurements of experimental acute lung injury in animals: an official American Thoracic Society Workshop report. Am J Respir Cell Mol Biol.

